# LC-MS/MS analysis reveals plasma protein signatures associated with lymph node metastasis in colorectal cancer

**DOI:** 10.3389/fimmu.2024.1465374

**Published:** 2024-10-23

**Authors:** Chunsong Pang, Fang Xu, Yingwei Lin, WeiPing Han, Nianzhu Zhang, Lifen Zhao

**Affiliations:** Department of Laboratory Medicine, The Second Hospital of Dalian Medical University, Dalian, Liaoning, China

**Keywords:** colorectal cancer, lymph node metastasis, plasma, biomarkers, proteomic

## Abstract

**Objectives:**

Colorectal cancer (CRC) is a major global health concern, ranking as the third most common cancer and the fourth leading cause of cancer-related deaths worldwide. Currently, the diagnostic accuracy of Lymph node metastasis (LNM) is currently unsatisfactory. Therefore, there is an urgent need to develop a reliable tool that can accurately predict lymph node metastasis (LNM) in patients diagnosed with CRC.

**Methods:**

We conducted an extensive proteomics investigation aimed at examining lymph node metastasis (LNM) in individuals diagnosed with colorectal cancer (CRC). In the discovery stage, employing a mass spectrometry-based proteomic approach, we analyzed a cohort of 60 colorectal cancer patients (NM=30, LNM=30), identifying distinct molecular profiles that differentiate patients with and without lymph node metastasis (LNM). Subsequently, we validated the protein classifier associated with lymph node metastasis.

**Results:**

We elucidated a combinatorial predictive protein biomarker that can distinguish patients with and without lymph node metastasis by LC-MS/MS. The classifier achieved an area under the curve (AUC) of 0.892 (95% CI, 0.842-0.941), while in the testing cohort, it attained an AUC of 0.929 (95% CI, 0.824-1.000). Furthermore, the four protein markers demonstrated an AUC of 0.84 (95% CI, 0.783–0.890) in the validation cohort. Additionally, we categorized patients into three types based on immunophenotyping. Type 1 primarily consisted of patients with negative lymph node metastasis (NM), characterized by immune cells such as NK cells, CD4 T effector memory cells, and memory B cells. Type 2 mainly included patients with positive lymph node metastasis (LNM), characterized by immune cells such as mesangial cells, epithelial cells, and mononuclear cells. In Type 1, a prominent upregulation observed in immune inflammation, as well as in glucose and lipid metabolism. In Type 2, significant upregulation was evident in pathways such as pyrimidine metabolism and cell cycle regulation. The findings of this study suggest that immune mechanisms may exert a pivotal role in the process of lymph node metastasis in CRC.

**Conclusions:**

Here, we present plasma protein signatures associated with lymph node metastasis in colorectal cancer (CRC). However, further validation across multiple centers is necessary to generalize these findings.

## Introduction

Colorectal cancer (CRC) is the third most commonly diagnosed cancer and the second leading cause of cancer-related deaths (1), with almost 900,000 deaths annually (2). It is estimated that by 2030, there will be over 2.2 million new cases and 1.1 million deaths due to CRC worldwide.

Lymph node metastasis (LNM) is the primary form of metastasis in colorectal cancer (CRC) and a significant contributor to postoperative recurrence and mortality. Accurate preoperative prediction of lymph node status in CRC is crucial for making appropriate therapeutic decisions. This includes the use of neoadjuvant and/or adjuvant chemotherapy for patients with LNM, or the implementation of a more conservative approach to minimize bowel resection for patients without LNM (3, 4).

Several histopathological findings, including lymphatic invasion, tumor depth, poor tumor differentiation, and tumor budding, are recognized as predictors of lymph node (LN) metastasis (5). However, these findings are only available postoperatively. Additionally, imaging techniques are recommended for monitoring LN metastasis in colorectal cancer (CRC) (6). Computed tomography (CT) and magnetic resonance imaging (MRI), which are commonly employed for the assessment of LN metastases, largely depend on the radiologist’s training and experience, as well as the quality of the imaging equipment (7). These mainstream imaging modalities **l**ack precision for certain assessment and diagnosis of LNs, often leading surgeons to perform major resection, which involves removing large parts of the intestines and healthy surrounding LNs, to avoid recurrence of the disease (8). Additionally, they are inappropriate for patients with implants or impaired renal function (9). Thus, the search for more precise and noninvasive biomarkers to predict LN metastasis in patients with CRC continues to be crucial. Accurate identification of lymph node (LN) involvement in patients with CRC is crucial for prognosis and treatment strategy decisions (10). Although several histopathologic findings, such as lymphatic invasion and tumor differentiation, are known to be predictors of LN metastasis, they are only available postoperatively (5). Preoperative knowledge of LN metastasis can provide valuable information for determining the need for adjuvant therapy and the adequacy of surgical resection, thus aiding in pretreatment decision making (11). Therefore, there is an urgent need to develop a tool capable of accurately predicting lymph node metastasis (LNM) in patients diagnosed with CRC.

Proteins, as executors of biological functions, received increasing attention from researchers. Recent proteomic investigations of CRC identified novel protein signatures, molecular subtypes, and metastasis markers as well as differences in carcinogenesis between right- and left-sided CRC (12–14). In fact, several studies have focused on accurately predicting lymph node metastasis in CRC. Koichiro et al. performed proteomic analysis using isobaric tags for relative and absolute quantification (iTRAQ). The methodology led to the identification of heat shock protein 47 (HSP47) as a novel predictor of CRC lymph node metastasis (15). Cheng et al., used two-dimensional difference gel electrophoresis MS/MS in colorectal cells (CRC), identified GSN and PRDX4 were lymph node metastasis (LNM)-associated proteins (16). Chao et al., characterized the immune infiltration landscape of CRC samples from The Cancer Genome Atlas (TCGA) and the Gene Expression Omnibus (GEO) databases, indicated FSTL3 is a potential immunotherapeutic target to block LNM for CRC (17). These studies unraveled a new paradigm for assessment and identification the LN metastasis in patients with CRC. However, these studies focused on CRC clinical tissue samples. Most recently, Yulin et al. developed urinary protein signatures for the diagnosis and metastatic risk stratification of colorectal cancer (CRC) (18). Circulating protein biomarkers have garnered increasing attention due to their ease of collection, cost-effectiveness, and suitability for repeated sampling (19). Extensive research has been conducted to identify blood-based biomarkers for the early detection and prognosis of CRC (20).

In the present study, we assembled two cohorts of patients with or without LNM and evaluated the changed plasma proteomics. We developed a high-performance predictive model that demonstrated superior diagnostic accuracy for predicting LNM in patients suffering from this aggressive malignancy.

## Methods

### Study design and patients

**Figure 1:**
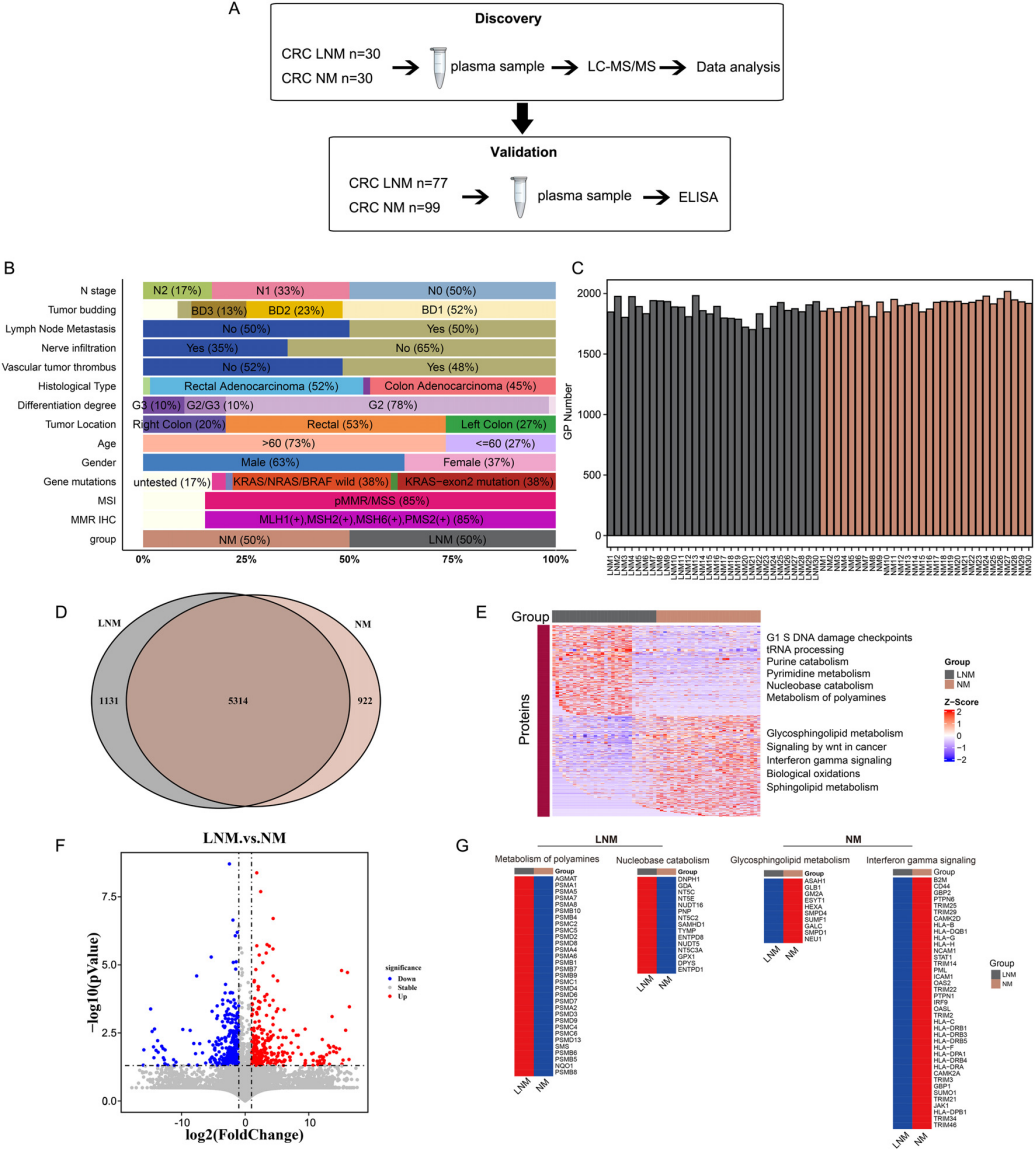
Experimental design and proteomic characteristics of CRC with or without lymph node metastasis were investigated. **(A)** CRC patients were stratified into two cohorts: a discovery cohort (N=60) and a validation cohort (N=176). A two-stage workflow, comprising LC-MS/MS and ELISA, was employed to establish a comprehensive and high-throughput plasma-based cancer biomarker method. **(B)** The clinical features of the discovery cohort patients. **(C)** The number of identified proteins in plasma samples from 30 patients with non-metastatic colorectal cancer (CRC NM) and 30 patients with metastatic colorectal cancer (CRC LNM) in the discovery cohort. **(D)** Venn diagram illustrates the intersection of significantly altered proteins in CRC NM and CRC LNM samples in the discovery cohort. **(F)** Volcano plot of LNM versus NM proteins. **(E, G)** Heat map of the molecular expression of proteins in the feature pathways of two sets of data.

The objective of this study was to systematically identify and validate plasma biomarkers for lymph node metastasis in colorectal cancer. The comprehensive workflow is depicted in **Figure 1A**. A total of 236 patients diagnosed with colorectal cancer were enrolled at the Second Affiliated Hospital of Dalian Medical University between January 2023 and March 2024. Pathological confirmation was obtained from two experienced pathologists, and plasma samples were collected prior to surgical intervention or initiation of radiotherapy and chemotherapy. In all enrolled patients, the inclusion criteria were as follows: age between 18 and 80 years old; having undergone curative surgical resection; and confirmed pathologically as colorectal adenocarcinoma, mucinous adenocarcinoma, or signet ring cell carcinoma according to the AJCC/UICC 8th edition TNM staging system. Exclusion criteria included patients who only received endoscopic treatment, were diagnosed with familial adenomatous polyposis, Lynch syndrome, or a history of inflammatory bowel disease, underwent transanal endoscopic microsurgery, had simultaneous infiltrating cancer, or had missing data. Patients who received chemotherapy or radiotherapy before surgery are also excluded.

### Sample collection

After obtaining patient consent, peripheral venous blood was collected and transferred into vacutainers containing sodium citrate as an anticoagulant. The samples were centrifuged at 2000g for 10 minutes after 30 min of collection. Subsequently, the resulting plasma was rapidly frozen using liquid nitrogen and stored at -80°C until further analysis.

### Plasma protein extraction and trypsin digestion

The plasma sample to be tested was melted at 4°C. Plasma samples were mixed with 100 μL 50 mM ammonium bicarbonate (ABC) buffer, and the proteins were inactivated at 95°C for 3 min. The samples were cooled to room temperature, digested using trypsin for 16 h in a 37°C incubator, then extracted, lyophilized and followed by desalinated. Finally, the collected peptides were added with 100 μL 0.1% FA for LC-MS/MS analysis.

### Mass spectrometry analysis

The plasma samples were subjected to LC-MS/MS, consisting of an EASY-nLC 1200 ultra-high-pressure system (Thermo Fisher Scientific) coupled via a nano-electrospray ion source to a Q Exactive HF-X Hybrid Quadrupole-Orbitrap mass spectrometer (all Thermo Fisher Scientific). Mobile phases A and B were 99.9/0.1% water/FA (v/v) and 80/20/0.1% ACN/water/FA (v/v/v). MS spectra were acquired with a Data-Independent Acquisition (DIA) method. The DIA-MS method consisted of an MS1 scan from 300 to 1400 m/z range (AGC target of 4 × 105, maximum injection time of 50 ms) at a resolution of 60,000 and 30 DIA segments (AGC target of 5 × 10^4^, maximum injection time of 22 ms) at a resolution of 15,000.

### Peptide identification and protein quantification

All data were processed using “Firmiana” (a one-stop proteomic cloud platform, https://phenomics.fudan.edu.cn/firmiana/). The data were search against UniProt human protein database (updated on 2019.12.17, 20406 entries) using FragPipe (v12.1) with MSFragger (2.2) (DIA data) and Mascot search engine (DDA data). The mass tolerances were: 20 ppm for precursor and 50 mmu for product ions collected by Q Exactive HF-X. Up to two missed cleavages were allowed. The database searching considered cysteine carbamidomethylation as a fixed modifcation, and N-acetylation, and oxidation of methionine as variable modifications. Precursor ion score charges were limited to +2, +3, and +4. For the quality control of protein identification, the target-decoy-based strategy was applied to confirm the FDR of both peptide and protein, which was lower than 1%. Quantification of identified peptides was calculated as the average of chromatographic fragment ion peak areas across all reference spectra libraries. Label-free protein quantifications were performed as previously reported with the iBAQ algorithm. The peak area values as parts of corresponding proteins were calculated. The fraction of total (FOT), defined as a protein’s iBAQ divided by the total iBAQ of all identified proteins within one sample, was used to represent the normalized abundance of a particular protein across samples. the FOT values were further multiplied by 105 for ease of presentation, and missing values were replaced by the minimal value.

### Differential protein analysis

The Student’s t-test was used to examine whether proteins were differentially expressed between NM and LNM. Upregulated or downregulated proteins are defined as proteins differentially expressed in one group compared with the other group (p < 0.05, Fold change >2 or <0.5).

### Pathway enrichment analysis

The differentially expressed proteins were subjected to enrichment analysis in Gene Ontology and KEGG pathways using DAVID (https://david.ncifcrf.gov/) and ConsensusPathwayDB (http://cpdb.molgen.mpg.de/), with a false discovery rate (FDR) threshold of less than 0.05. Pathways were determined by utilizing gene sets from the KEGG, Reactome, and GO database.

### Weighted gene correlation network analysis

To identify gene modules exhibiting differential co-expression, we employed Weighted Gene Correlation Network Analysis (WGCNA) on the proteins expressed in patient samples. The analysis was conducted using the WGCNA package in R. Module eigenproteins were determined by calculating the first principal components of co-expressed genes within each module. The eigengenes were subsequently utilized to assess the association between a gene module and clinical information. The degree of correlation between a gene and other members within its respective gene module was quantified using eigengene-based connectivity (kME).

### Enzyme-linked immunosorbent assay (ELISA)

ACTR1B levels were determined using enzyme-linked immunosorbent assays (ELISAs) (abx385606, Abbexa Ltd., Cambridge, United Kingdom). KIF5B (ELK0377), NAXE (ELK4941), and RBM3 (ELK4844) levels were determined using ELISA kit (ELK Biotechnology, China). All assays strictly adhered to the manufacturers’ instructions. Each sample was measured twice, and in all measurements, the intra-assay coefficient of variation remained below the threshold specified by the manufacturers. The standard curve was generated using Curve Expert 1.4 software, while sample concentrations were determined through data analysis in Microsoft Excel.

### Statistical analysis

The statistical analysis was performed using GraphPad Prism 8.0 (San Diego, CA, USA) and R software. Proteins that met the criteria of p-values < 0.05 and fold changes > 1.5, or other specified thresholds, were visualized using the heatmaps package in R. Between-group comparisons of proteins were conducted using paired two-class comparison in R with a false discovery rate (FDR) threshold of 0.05. Pathway enrichment analysis was conducted to identify pathway alterations using the Reactome database. Differential analysis was conducted between samples with lymph node metastasis (LNM) and without lymph node metastasis (NM) using Fisher’s exact t-tests for protein comparisons. Correlation analysis was conducted using the Spearman rank test, while quantitative results from ELISA data were analyzed by the Mann-Whitney rank test in GraphPad Prism 8.0 software. n.s means not significant. *, **, **** and **** represent p-value less than 0.05, 0.01, 0.001 and 0.0001, respectively.

## Results

### Cohort characteristics and research design

To identify the mechanisms of lymph node metastasis (LNM) and the associated protein signatures in colorectal cancer (CRC), we conducted mass spectrometry (MS)-based proteomics to analyze plasma samples from patients with lymph node metastasis negative (NM) and lymph node metastasis positive (LNM) CRC. The discovery cohort comprised 60 patients, while the validation cohort included 176 patients. The overall workflow of this study is presented in **Figure 1A** (**Supplementary Figure 3A**). The clinicopathologic characteristics of the participants (discovery cohort) are summarized in **Figure 1B**. LNM is closely associated with N stage (p < 0.001, Pearson’s chi-squared test), nerve infiltration (p < 0.001, Pearson’s chi-squared test), vascular tumor thrombus (p < 0.001, Pearson’s chi-squared test), and gender (p < 0.05, Pearson’s chi-squared test). Detailed P-value results are presented in **Supplementary Table 1**. In agreement with previous reports, LNM patients were most staged in N1 and N2 stages. However, no significant correlation was observed between LNM and histological type, tumor differentiation, gene mutations, or tumor location. It has been reported that LNM is correlated with tumor location and poor differentiation degree in CRC FFPE tissues (21). However, no significant correlation was observed between LNM and histological type, tumor differentiation, gene mutations, or tumor location in our study. The occurrence of this phenomenon may be linked to the specific type of specimen being investigated and the geographical region in which it is found.

### Discovery of differential plasma proteins using LC-MS/MS approach

First, plasma protein candidates were discovered by high-resolution LC-MS/MS on a Q Exactive HF-X mass spectrometer. Overall, more than 7000 protein groups were identified by using the criteria of a 1% false discovery rate (FDR) at both the peptide and protein levels, with a range 1700 to 2100 protein groups per NM and LNM plasma samples (**Figures 1C**). Proteome quantification was carried out utilizing the intensity-based absolute quantification (iBAQ) algorithm. The abundance of the identified proteins varied widely, spanning approximately about eight orders of magnitude (**Supplementary Figures 1A, B**). In addition, the consistency of the of the samples within the group was assessed using Spearman correlation coefficients. The Pearson’s correlation coefficient, calculated for NM and LNM samples, was more than 0.89 and 0.82, respectively, indicating that the MS data were of high quality (**Supplementary Figures 1C, D**). Intersection analysis with a Venn diagram of all proteins associated with LNM and NM exhibited 5300 shared proteins (**Figure 1D**). Meanwhile, 1131 proteins specifically expressed in LNM group and 922 proteins specifically expressed in NM group. Further filtered the data, we found 300 proteins were significantly upregulated, whereas 309 proteins were downregulated expressed in LNM patients (with a log2-fold change [log2FC]>1 or <-1 and p<0.05, Student’s t-test) (**Figure 1F**). GSVA analysis was performed to analyze the underlying pathways associated with LNM. G1 S DNA damage checkpoints, tRNA processing, Purine catabolism, Pyrimidine metabolism, Nucleobase catabolism and Metabolism of polyamines pathways were found to be enriched in the LNM group (**Figure 1E**). Metabolism of polyamines pathway related proteins (AGMAT, PSMA1, PSMA5 et al) and Nucleobase catabolism pathway related proteins (DNPH1, GDA, NT5C et al) were up-regulated in the LNM group (**Figure 1E**). On the other hand, glycosphingolipid metabolism, signaling by Wnt in cancer, interferon gamma signaling, biological oxidations, and sphingolipid metabolism were enriched in the NM group. Proteins related to the glycosphingolipid metabolism pathway (ASAH1, GLB1, GM2A, etc.) and proteins related to the interferon gamma signaling pathway (B2M, CD44, GBP2, etc.) were up-regulated in the NM group (**Figure 1G**). These results suggest that various signaling and metabolic pathways may contribute to lymph node metastasis in colorectal cancer.

### Inter-group differences in pathway representation and clinical features associated with proteomic profiles

**Figure 2:**
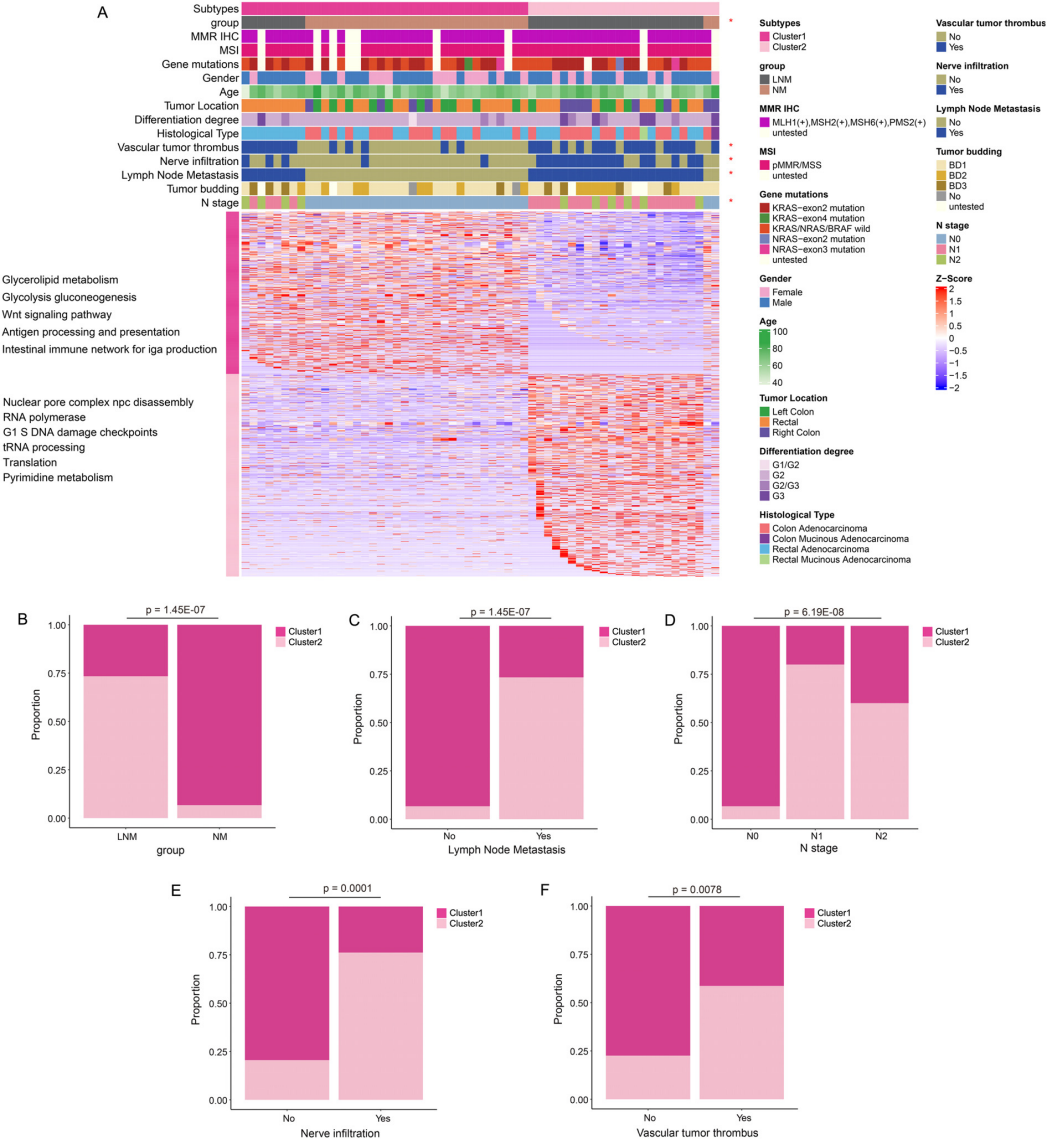
Protein molecular subtypes and their clinicopathological features. **(A)** Identification of protein subtypes (k=2), clinical pathological characteristics, and enriched pathways was conducted through consensus clustering analysis in the discovery cohort. **(B–F)** The percentage bar chart illustrated the clinical features and metrics that exhibited significant correlation with the protein subtypes.

Consensus Cluster Plus analysis (**Figure 2A**) of the proteins identified two distinct patient clusters: Cluster 1 and Cluster 2. Cluster 1 primarily consisted of samples from NM patients, while Cluster 2 predominantly comprised samples from LNM patients. The analysis revealed that clinical features, including group classification, lymph node metastasis, N stage, nerve infiltration, and vascular tumor thrombus, were significantly associated with protein classification. The percentage bar chart (**Figures 2B–F**) showed the proportion of Cluster 2 in LNM group was high, while the proportion of Cluster 1 in NM group was high. The proportion of Cluster 2 was significantly higher in patients with Lymph Node Metastasis, Vascular tumor thrombus or Nerve infiltration positive compared with negative patients. The ratio of cluster 1 in N0 stage patients was high, while the ratio of Cluster 2 in N1/N2 stage was high. Moreover, Cluster 1 was chiefly enriched for proteins with glycolipid metabolism, glycolysis, WNT signaling, antigen processing and presentation, Intestinal immune network. Cluster 2 was enriched for proteins with nuclear pore complex npc disassembly, RNA polymerase, G1 and S phase checkpoint, tRNA processing, translation.

### WGCNA and clinical correlation analysis

**Figure 3:**
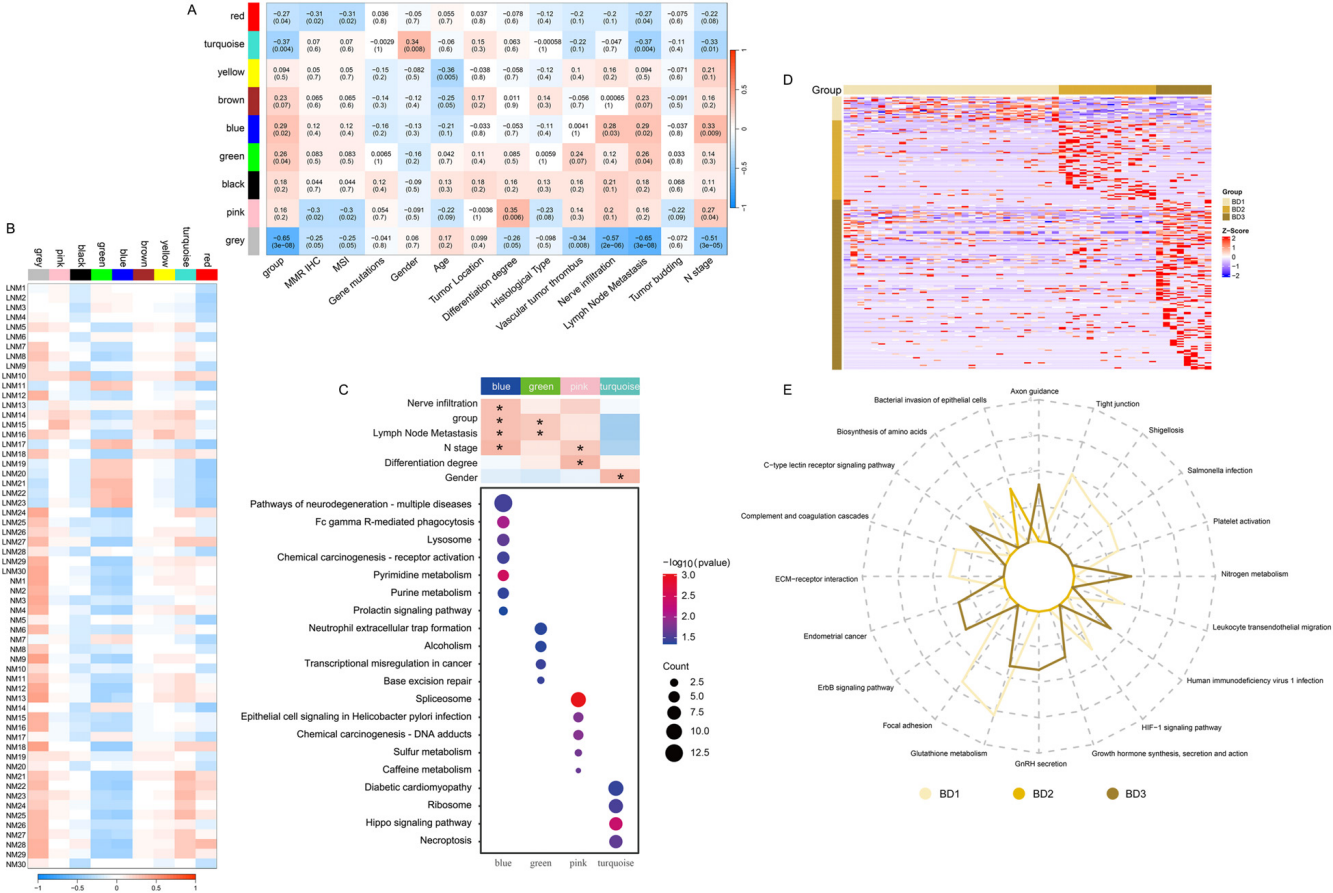
Identification of protein modules with high correlation using WGCNA and evaluation of their associations with clinical variables. **(A)** Correlation between module eigenvalues and diverse phenotypic traits. **(B)** Correlation between module eigenvalues and diverse samples. **(C)** Enrichment analysis of WGCNA module pathway. **(D)** Heat map illustrating the proteins significantly associated with tumor budding. **(E)** The radar diagram illustrating the categorization of pathways. The length of the line at each point position represents the number of pathways in corresponding categories.

The proteins and continuous clinical variables were used to perform weighted gene correlation network analysis (WGCNA) (Methods), which was an unsupervised manner to identify groups of co-regulated proteins and the association with clinical variables. Nine modules were identified. The analysis of module-trait relationships revealed that nerve infiltration, group, lymph node metastasis, and N stage were significantly positively correlated with the blue module (**Figure 3A**). Additionally, both group and lymph node metastasis exhibited a positive correlation with the green module. Gender demonstrated a significant positive correlation with the turquoise module, while the differentiation stage was significantly positively correlated with the pink module. Furthermore, the pathways enriched in the modules are presented in **Figure 3C**. The patient samples associated with the blue and green modules primarily consisted of LNM patients (**Figure 3B**). The proteins in the blue module were predominantly involved in pyrimidine metabolism, purine metabolism, Fc gamma receptor-mediated phagocytosis, and lysosomal function. In contrast, the proteins in the green module were mainly engaged in transcriptional regulation. The proteins in the pink module were enriched in spliceosome activity, chemical carcinogenesis, and coffee metabolism. Additionally, the proteins in the turquoise module were enriched in ribosomal function and Hippo signaling pathways (**Figure 3C**). Moreover, the heat map (**Figure 3D**) was utilized to visually represent the expression of significantly different proteins across the tumor budding groups BD1, BD2, and BD3. The pathways associated with tight junctions, focal adhesion, platelet activation, and extracellular matrix (ECM) receptor interactions were significantly enriched in the BD1 group (**Figure 3E**). The bacterial invasion of epithelial cells pathway was particularly enriched in the BD2 group. Additionally, the ERBB signaling, endometrial cancer, and axon guidance pathways were significantly enriched in the BD3 group.

### Protein tissue origin discovery

**Figure 4:**
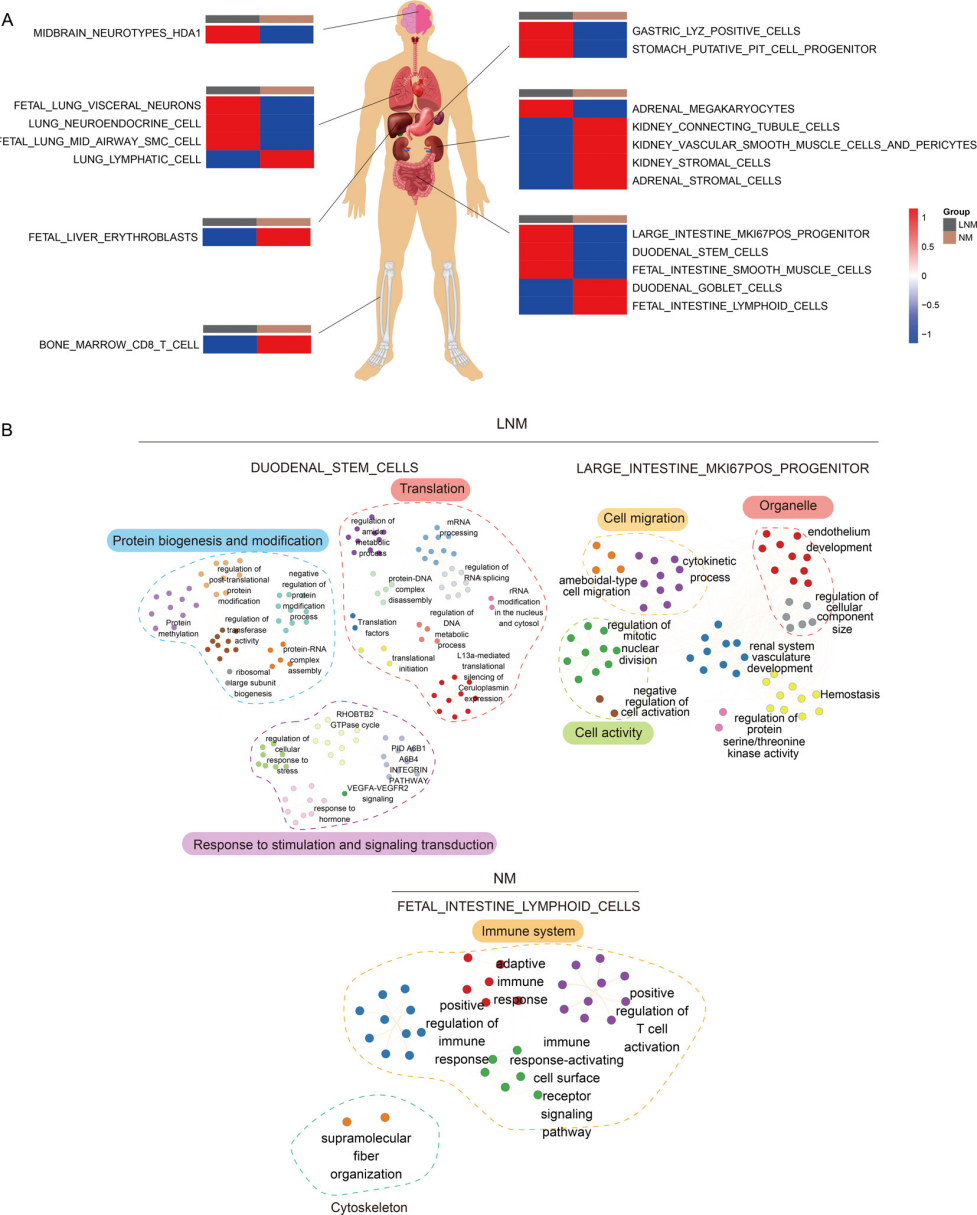
Analysis of tissue traceability. **(A)** Display of cellular damage across diverse organs. **(B)** The diagram of the network illustrating pathway interactions elucidates the interplay between various cellular pathways within the intestinal context.

To better analyze the reactions of various human organs, proteomic traceability was conducted. The tissue tracing method involves the use of individual cell data from academic literature and a database of human tissue-specific proteins (tissue enhanced proteins, HPA) to compile a set of proteins specific to different organs in the human body. This collection is subsequently employed to determine the significance of particular proteins in organ cells across diverse patient cohorts, yielding a metric for the state of the organ cells. This metric may serve as an indicator of the degree of cellular impairment within the patient’s organs. As shown in **Figure 4A**, the tissue traceability analysis revealed the impact of Lymph Node Metastasis (LNM) and Non-Metastasis (NM) on various organs including the brain, lung, stomach, intestine, liver, and bone marrow of the patients. In the LNM group, the functionality of nerves in the brain, neuroendocrine cells in the lungs, progenitor cells in the stomach, and stem cells in the intestines was impaired. Conversely, in the NM group, damage was observed in red blood cells in the liver, CD8 T cells in the bone marrow, stromal cells in the kidneys, and lymph node cells in the small intestine. According to the objective of the study, which is to determine lymph node metastasis in colorectal cancer, we selected gut-related cells that could be damaged in lymph node metastatic colorectal cancer and non-metastatic colorectal cancer. We used the highly expressed proteins from these cells to enrich the characteristic pathways and further demonstrated the interaction pathways of these enriched characteristic pathways. This suggests which characteristic pathways may promote cancer in colorectal cancer patients with and without lymph node metastasis. The pathway interaction network diagram (**Figure 4B**) illustrated the interactions between pathways in different types of cells in the intestine. In the LNM group, the interaction between major pathways of progenitor cells and stem cells in the intestine is primarily demonstrated. In the NM group, mutual regulation of major pathways in intestinal lymphocytes was primarily demonstrated, corresponding to the apparent activation of immunity in NM patients in the immune subtype mentioned below.

### Analysis of the correlation between immune phenotypes and clinical characteristics

**Figure 5:**
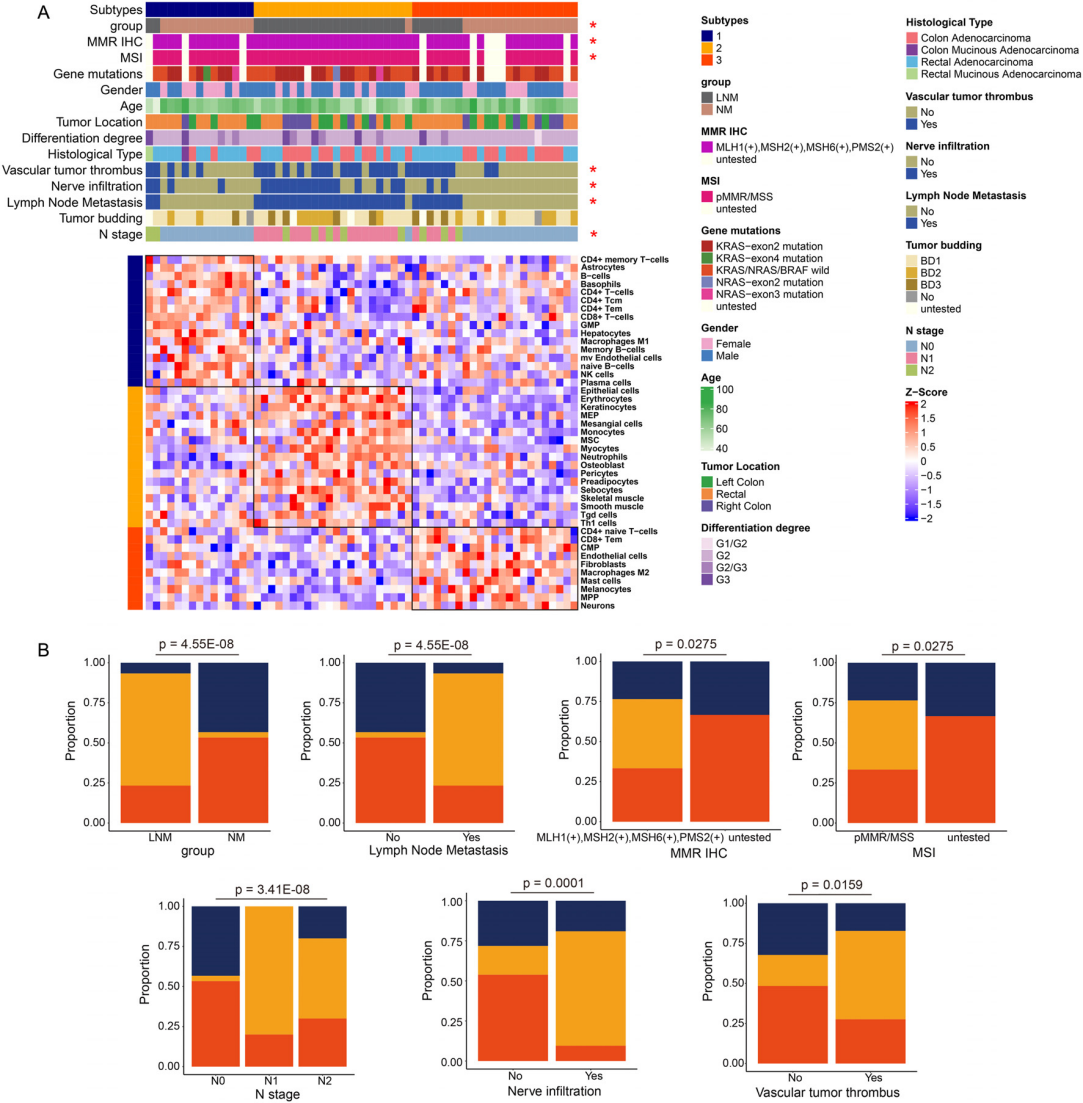
Analysis of immune phenotyping **(A)** Patients were categorized into three types based on their immunological profiles. **(B)** The percentage bar chart illustrates the clinical features and metrics that exhibited significant correlation with the protein classification.

As illustrated in **Figure 5A**, all patients are categorized into three types based on immune classification, with the immune characteristics of each type clearly depicted. Type 1 primarily includes patients with NM patients, characterized by a predominance of immune cells such as natural killer (NK) cells, CD4+ effector memory T (Tem) cells, and memory B cells. Type 2 predominantly corresponds to LNM patients, where the immune cell composition mainly consists of mesangial cells, epithelial cells, and monocytes. Type 3 encompasses both NM and LNM patients, whose immune cell profiles are primarily composed of CD4+ T cells, mast cells, and fibroblasts. Notably, the clinical features that show significant correlations with immune typing include group classification, mismatch repair (MMR) immunohistochemistry (IHC), microsatellite instability (MSI), the presence of vascular tumor thrombus, nerve infiltration, lymph node metastasis, and N stage. The percentage bar chart (**Figure 5B**) displayed clinical features and indicators that are significantly correlated with protein typing. They included group, lymph node metastasis, N stage, nerve infiltration, vascular tumor thrombus, MMR IHC, and MSI. For example, the proportion of immunotype 2 was the highest in the LNM group, while the proportion of immunotype 3 was the highest in the NM group. Scatter plots (**Supplementary Figure 2**) illustrated the pathways that are significantly correlated with the immune cells of the three immunophenotype types, which are listed from top to bottom as Type 1, Type 2, and Type 3. The red dots indicated pathways that exhibit significant correlations. Each dot on the left represented a negatively correlated pathway, highlighting the inhibitory effect of the immune microenvironment on the cellular function of that pathway. Conversely, each dot on the right represented a positively correlated pathway, suggesting that the immune microenvironment enhances the cellular function of the corresponding pathway. The heatmap illustrates the pathways that exhibit significant positive correlations with Type 1, Type 2, and Type 3, along with the expression levels of specific molecules within these pathways. The intensity of color on the heatmap corresponds to the expression level of the respective molecule depicted on the right side. A deeper red color indicates higher expression levels, while a deeper blue color signifies lower expression levels or absence of expression for the molecule. The analysis of overall molecular expression changes within the pathways revealed a notable upregulation in Type 1, particularly in pathways associated with immune inflammation, as well as glucose and lipid metabolism. In Type 2, significant upregulation was observed in pathways such as pyrimidine metabolism and the cell cycle. For Type 3, the pathways that exhibited significant upregulation included immune response, DNA replication, and glucose and lipid metabolism.

### Machine learning–based selection of combinatorial biomarkers for classification of CRC with LNM

**Figure 6:**
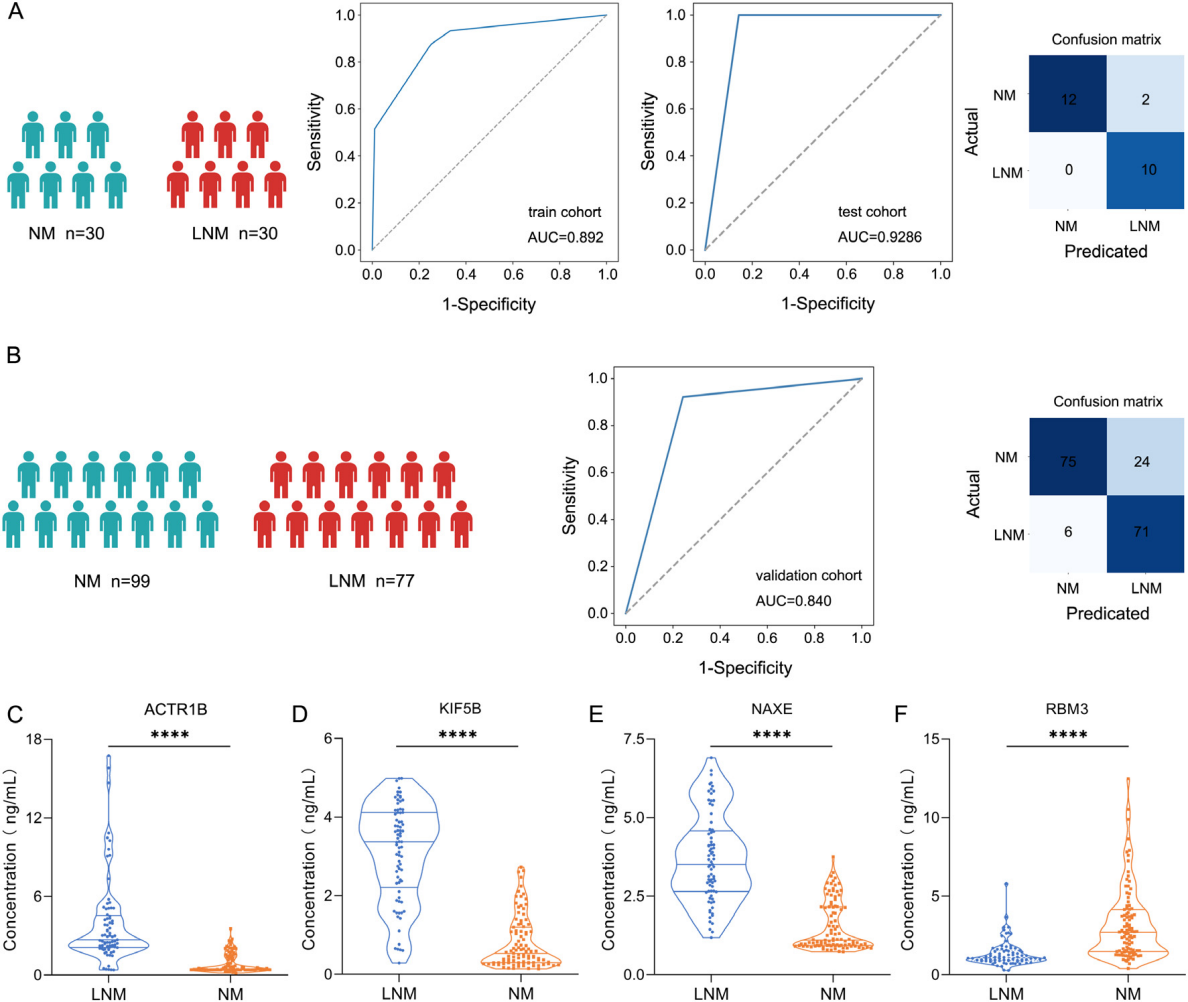
Screening protein biomarkers for lymph node metastasis of colorectal cancer using machine learning techniques. **(A)** Receiver operating characteristic (ROC) curve for the classification model in the discovery cohort (train cohort and test cohort). Classification confusion matrix of the classifier in the test cohort. **(B)** ROC curve for the classification model in the validation cohort. Classification confusion matrix of the classifier in the validation cohort. **(C-F)** The plasma concentrations of ACTR1, KIF5B, NAXE, and RBM3 by ELISA. **** represent p-value less than 0.0001.

To identify protein markers that can predict lymph node metastasis in patients with colorectal cancer (CRC), we developed a classifier that effectively distinguishes between LNM and non-LNM patients, thereby enhancing clinical decision-making for CRC management. Candidate biomarkers were selected from proteins that exhibited significant differential expression (|log2(FC)| > log2(1.5)). The 60 patients were divided into a training cohort (LNM N=18, NM N=18) and a test cohort (LNM N=12, NM N=12). The Least Absolute Shrinkage and Selection Operator (LASSO) logistic regression was employed to assess feature importance (significance of the predictive features). The LNM status was used to evaluate the discriminative power of each signature. This process generated a classifier, including ACTR1B, KIF5B, NAXE, and RBM3 (**Supplementary Tables 2**–**4**), which facilitated accurate discrimination between LNM and NM patients with CRC. The mass spectrometry results for these four proteins were present in **Supplementary Figure 3B**. Through fivefold cross-validation, the classifier in the training cohort achieved an AUC of 0.892 (95% CI, 0.842-0.941) (**Figure 6A**), while in the testing cohort, the classifier achieved an AUC of 0.929 (95% CI, 0.824-1.000), which indicated the classifier is an effective predictor for lymph node metastases in CRC. To further assess the predictive power of the proteomic classifier in the clinic, a validation cohort consisting of 176 patients (LNM n=77, NM n=99) was utilized (**Figure 6B**). The plasma concentrations of the four proteins were measured using ELISA. Consistent with the proteomics findings, the plasma concentrations of ACTR1B (P<0.05), KIF5B (P<0.05), and NAXE (P<0.05) showed a general increase in LNM patients (Student’s t-test) (**Figures 6C–E**). Conversely, RBM3 (P<0.05) (**Figure 6F**) exhibited a significant increase in NM patients according to the ELISA data (Student’s t-test). Moreover, the four-protein classifier effectively distinguished between LNM and NM patients, achieving an AUC of 0.84 (95% CI, 0.783–0.890). In addition, the AUC values for the four proteins demonstrate robust performance (**Supplementary Figure 3D**). Furthermore, we performed decision curve analysis (DCA) to evaluate the clinical utility of our established protein classifier by estimating net benefits across various threshold probabilities. The DCA curves demonstrated that the probability of net benefit varied between 0% and 80% within the validation cohort, indicating that our model possesses significant potential to improve predictions of lymph node metastasis in CRC (**Supplementary Figure 3C**).

In summary, our plasma four-protein classifier can more accurately differentiate between LNM and NM CRC patients, providing better guidance for clinical decision-making.

## Discussion

CRC is a significant global health concern, ranking as the third most common cancer and the fourth leading cause of cancer-related deaths worldwide (22). Lymph node metastasis (LNM) plays a crucial role in the spread of CRC, influencing treatment decisions, chemotherapy plans, and patient survival rates (23). Accurate assessment of lymph node status is essential in CRC to customize personalized treatment strategies, known as precision medicine, and improve individualized patient care. Diagnostic imaging is essential for the diagnosis, staging, and monitoring of treatment in individuals with CRC. Currently, computed tomography (CT) and magnetic resonance imaging (MRI) are primarily used for diagnosing lymph node metastasis based on the size, shape, and structure of lymph nodes. However, the diagnostic accuracy of LNM is currently unsatisfactory (24). For diagnosing LNM, preoperative invasive lymph node biopsy and pathology were the conventional gold standard (40). However, there are several drawbacks, such as invasiveness, high cost, inter-observer variation, and susceptibility to sampling errors (25).

Liquid biopsy assays are gaining momentum in the field of cancer patient management, primarily for improved and earlier disease detection, monitoring disease progression after treatment, as well as for expedited drug development (26). Liquid biopsy, which initially focused on plasma proteins, has now expanded to encompass a diverse array of molecules and circulating cells. Although early challenges were encountered in cancer diagnostics using protein assays, recent advancements have significantly improved our understanding of the protein profiles associated with various cell types in the body. This knowledge has facilitated the identification of specific proteins that show promise as diagnostic targets (27). Moreover, technological advancements in protein assays are enhancing their performance, raising expectations for their potential use in screening for cancer and monitoring therapy responses in common malignancies. The significance of improved cancer diagnostics through protein assays is substantial. Early detection can prevent metastasis, potentially saving lives, reducing healthcare expenses, extending productive years, and fostering growth in the diagnostic testing industries (28).

In this study, we conducted a comprehensive proteomic investigation aimed at examining lymph node metastasis (LNM) in individuals diagnosed with CRC. During the discovery stage, we employed a mass spectrometry-based proteomic approach to analyze a cohort of 60 colorectal cancer patients, identifying distinct molecular profiles that differentiate those with lymph node metastasis from those without. Furthermore, we elucidated a combinatorial predictive protein biomarker capable of distinguishing between patients with and without lymph node metastasis. Based on the CRC proteomics dataset, we developed and validated a protein signature model consisting of four proteins. The classifier achieved an AUC of 0.892 (95% CI, 0.842-0.941), while in the testing cohort, the classifier achieved an AUC of 0.929 (95% CI, 0.824-1.000). Moreover, the four-protein markers achieved an AUC of 0.84 (95% CI, 0.783–0.890) in the validation cohort. Intriguingly, the protein ACTR1B has been identified as a human brain protein associated with the perception of bitter or sweet beverages (29). Activin A regulates fibroblast-mediated collagen gel contraction by binding to a cell-surface receptor complex comprising two distinct types of receptors, namely activin type I receptor (specifically ActR-IB) and type II receptor (ActR-II) (30, 31). KIF5B, a member of the Kinesin-1 family, has been extensively investigated and found to play crucial roles in diverse biological processes, including myogenesis, nuclear transport, kidney development, chondrocyte differentiation, viral replication, and tumor progression (32–35). Recently, KIF5B has been identified as a promising early biomarker in the advanced stage of pancreatic cancer. RBM3 was able to perfectly discriminate human high-grade astrocytomas/glioblastomas and control tissues (36). In addition, the downregulation of RBM3 has been documented to induce synaptic loss (37), leading to neurodegeneration and thus representing a promising therapeutic target for Lewy body dementia (LBD) (38). The present study identified these four proteins as the most crucial indicators for distinguishing between LNM and NM, demonstrating exceptional specificity and sensitivity. Recent research has demonstrated a strong association between pyrimidine synthesis and cancer (39, 40). The rapid proliferation of CRC cells necessitates heightened nucleotide biosynthesis to support cell growth (41). Inhibiting pyrimidine biosynthetic genes at the molecular and pharmacological levels has been shown to impede liver metastatic colonization in CRC (42). Consequently, proteins involved in nucleotide metabolism represent promising targets for the treatment of CRC (43). Recently, an increasing body of research has revealed that transfer RNA (tRNA) can undergo enzymatic cleavage, leading to the generation of tRNA-derived fragments (tRFs). These fragments participate in cell apoptosis (44), tumor growth, and M2 macrophage polarization (45) in CRC.

In this study, intergroup comparative analysis revealed that, at the protein level, the characteristic pathways of the LNM group primarily included the G1/S cell cycle, tRNA processing, and purine and pyrimidine metabolism pathways. Kevin Brennan discovered that the initiation of LNM in head and neck cancer is triggered by the absence of p53-DREAM-mediated suppression of G1/S phase cell cycle genes during the early stages of tumor development (46). Additionally, TEFM was found to enhance the growth and spread of hepatocellular carcinoma (HCC) by facilitating the transition from the G1 to S phase (47). Furthermore, SLC12A5 was shown to significantly promote the G1/S cell cycle transition and enhance lung metastasis in colorectal cancer (48).

While our findings demonstrate promising potential, it is crucial to acknowledge certain limitations. Firstly, the traditional diagnostic biomarkers serum carcinoembryonic antigen (CEA), glycoconjugate antigen (CA) 19-9 and CA72-4 levels were not included in the study. Previous studies have demonstrated a potential correlation between these factors and the presence of lymph node metastasis in stage T1 colorectal cancer (49). We have noticed this issue. Unfortunately, the patient cohorts analyzed in our study had limited availability of tumor marker data. In future investigations, we will integrate these serologic tumor markers with our blood proteome signature to further investigate their potential in enhancing the predictive accuracy of lymph node metastasis. Second, our study included patients with T1-T4 stage. In recent studies, there has been a greater emphasis on investigating the T1 and T2 stage. In view of this fact, we will collect more T1 stage plasma samples for further research. Third, the clinical cohorts examined for biomarker training and validation in this study were relatively small. Therefore, it may be imperative to conduct a future prospective study involving larger patient cohorts from multiple centers to substantiate the clinical significance of the identified biomarkers.

In conclusion, we present plasma protein signatures associated with lymph node metastasis in CRC, although further validation across multiple centers is necessary to generalize these findings.

## Data Availability

The datasets presented in this study can be found in online repositories. The names of the repository/repositories and accession number(s) can be found below: iProx under project ID IPX0009289000.

## Supplementary material

Supplementary Table 1 The clinicopathologic characteristics of the participants (discovery cohort).

Supplementary Table 2 Diagnostic model construction.

Supplementary Table 3 Diagnostic model construction-all samples.protein.

Supplementary Table 4 Diagnostic model construction-Selected Featrues.

Supplementary Figure 1 Protein abundance and correlation analysis among samples. **(A)** Cumulative protein identification counts across all samples. **(B)** Protein Quantitative Abundance Density Distribution Chart. **(C)** Correlation analysis among the LNM group samples. **(D) **Correlation analysis among the NM group samples.

Supplementary Figure 2 Pathways significantly associated with immune subtyping characteristics. **(A–C)** Scatter plots depict the pathways exhibiting significant correlations with immune cells in immunophenotyping (A cluster 1, B cluster 2, and C cluster 3) (left). The heatmap revealed significant positive correlations between immunophenotyping and the expression of specific molecules within the pathways (right).

Supplementary Figure 3
**(A)** Flow chart of the study. **(B)** The mass spectrometry results for these four proteins. (C) DCA for assessing the clinical utility of our established protein classifier. (D) The ROC curve analysis for the four proteins detected using ELISA.
