# Supplementary material for: LC-MS/MS analysis reveals plasma protein signatures associated with lymph node metastasis in colorectal cancer
Source: Front Immunol. 2024 Oct 23;15:1465374. doi: 10.3389/fimmu.2024.1465374 (PMC11538601; doi:10.3389/fimmu.2024.1465374)
Supplement: Supplementary Table 1 — The clinicopathologic characteristics of the participants (discovery cohort). [file Table1.docx]

**Table1** The clinicopathologic characteristics of the participants (discovery cohort)

| **Characteristics** | **LNM** | **NM** | **Overall** | **P-value** |
| --- | --- | --- | --- | --- |
| N | 30 | 30 | 60 |  |
| MMR_IHC |  |  |  | 0.148 |
| MLH1(+), MSH2(+), MSH6(+), PMS2(+) | 28 (93.33%) | 23 (76.67%) | 51 (85.00%) |  |
| untested | 2 (6.67%) | 7 (23.33%) | 9 (15.00%) |  |
| MSI |  |  |  | 0.148 |
| pMMR/MSS | 28 (93.33%) | 23 (76.67%) | 51 (85.00%) |  |
| untested | 2 (6.67%) | 7 (23.33%) | 9 (15.00%) |  |
| Gene_mutations |  |  |  | 0.586 |
| KRAS/NRAS/BRAF wild | 12 (40.00%) | 11 (36.67%) | 23 (38.33%) |  |
| KRAS-exon2 mutation | 13 (43.33%) | 10 (33.33%) | 23 (38.33%) |  |
| NRAS-exon2 mutation | 1 (3.33%) | 0 (0.00%) | 1 (1.67%) |  |
| NRAS-exon3 mutation | 1 (3.33%) | 1 (3.33%) | 2 (3.33%) |  |
| KRAS-exon4 mutation | 0 (0.00%) | 1 (3.33%) | 1 (1.67%) |  |
| untested | 3 (10.00%) | 7 (23.33%) | 10 (16.67%) |  |
| Gender |  |  |  | 0.032 |
| Female | 7 (23.33%) | 15 (50.00%) | 22 (36.67%) |  |
| Male | 23 (76.67%) | 15 (50.00%) | 38 (63.33%) |  |
| Age |  |  |  | 0.243 |
| >60 | 10 (33.33%) | 6 (20.00%) | 16 (26.67%) |  |
| <=60 | 20 (66.67%) | 24 (80.00%) | 44 (73.33%) |  |
| Tumor_Location |  |  |  | 0.472 |
| Left Colon | 6 (20.00%) | 10 (33.33%) | 16 (26.67%) |  |
| Rectal | 18 (60.00%) | 14 (46.67%) | 32 (53.33%) |  |
| Right Colon | 6 (20.00%) | 6 (20.00%) | 12 (20.00%) |  |
| Differentiation_degree |  |  |  | 0.145 |
| G1/G2 | 0 (0.00%) | 1 (3.33%) | 1 (1.67%) |  |
| G2 | 21 (70.00%) | 26 (86.67%) | 47 (78.33%) |  |
| G2/G3 | 4 (13.33%) | 2 (6.67%) | 6 (10.00%) |  |
| G3 | 5 (16.67%) | 1 (3.33%) | 6 (10.00%) |  |
| Histological_Type |  |  |  | 0.516 |
| Colon Adenocarcinoma | 12 (40.00%) | 15 (50.00%) | 27 (45.00%) |  |
| Rectal Adenocarcinoma | 17 (56.67%) | 14 (46.67%) | 31 (51.67%) |  |
| Rectal Mucinous Adenocarcinoma | 1 (3.33%) | 0 (0.00%) | 1 (1.67%) |  |
| Colon Mucinous Adenocarcinoma | 0 (0.00%) | 1 (3.33%) | 1 (1.67%) |  |
| Vascular_tumor_thrombus |  |  |  | <0.001 |
| yes | 23 (76.67%) | 6 (20.00%) | 29 (48.33%) |  |
| no | 7 (23.33%) | 24 (80.00%) | 31 (51.67%) |  |
| Nerve_infiltration |  |  |  | <0.001 |
| yes | 19 (63.33%) | 2 (6.67%) | 21 (35.00%) |  |
| no | 11 (36.67%) | 28 (93.33%) | 39 (65.00%) |  |
| Lymph_Node_Metastasi |  |  |  | <0.001 |
| yes | 30 (100.00%) | 0 (0.00%) | 30 (50.00%) |  |
| no | 0 (0.00%) | 30 (100.00%) | 30 (50.00%) |  |
| Tumor_budding |  |  |  | 0.101 |
| BD 1 | 12 (40.00%) | 19 (63.33%) | 31 (51.67%) |  |
| BD 2 | 7 (23.33%) | 7 (23.33%) | 14 (23.33%) |  |
| BD 3 | 7 (23.33%) | 1 (3.33%) | 8 (13.33%) |  |
| no | 4 (13.33%) | 3 (10.00%) | 7 (11.67%) |  |
| N_stage |  |  |  | <0.001 |
| N0 | 0 (0.00%) | 30 (100.00%) | 30 (50.00%) |  |
| N1 | 20 (66.67%) | 0 (0.00%) | 20 (33.33%) |  |
| N2 | 10 (33.33%) | 0 (0.00%) | 10 (16.67%) |  |
