# Supplementary material for: LC-MS/MS analysis reveals plasma protein signatures associated with lymph node metastasis in colorectal cancer
Source: Front Immunol. 2024 Oct 23;15:1465374. doi: 10.3389/fimmu.2024.1465374 (PMC11538601; doi:10.3389/fimmu.2024.1465374)
Supplement: Supplementary Table 2 — Diagnostic model construction. [file Table2.docx]

**Diagnostic model construction**

1. Data pre-screening: the molecules with |log2(FC)| > log2(1.5) and pvalue < 0.05 (significance test by t.test) were selected.

2. Pre-screening: the cohort was randomly divided into training set and test set according to 6:4. The training set was based on the combination idea of multiple feature selection methods, combined with the weights obtained in each feature selection method, and the comprehensive weight value was calculated for the molecules frequently selected in the multi-feature selection method.

3. Biomarkers selection: according to the comprehensive weight value from large to small, add the candidate markers to the cohort one by one, calculate the AUC value, and select the cohort with the maximum AUC value as the candidate Biomarkers.


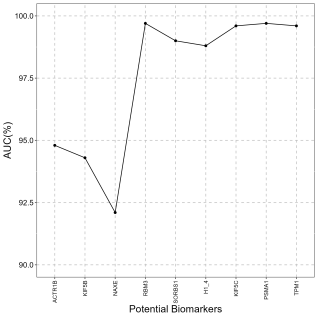


4. Validation of candidate Biomarkers: machine learning models, including logistic regression, random forest and support vector machine, were used to verify the results of the above screening


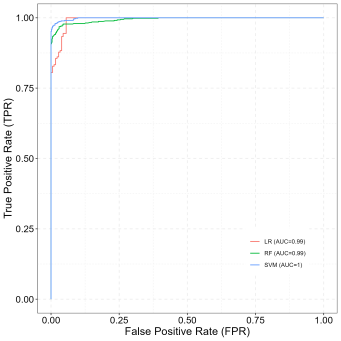


5. Evaluation of candidate Biomarkers: the main comparison is the ability to distinguish the two groups, which is shown as the expression heatmap of biomarkers in the experimental group and the control samples, and the lower the correlation between biomarkers, the smaller the fitting

6. Diagnostic panel model construction: The biomarker diagnostic panel model is constructed by logistic regression algorithm to obtain the regression coefficients and intercepts of Biomarkers, which are used to calculate the probability value p
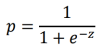
, where z= intercept + each Biomarker expression * regression coefficient. Youden index was used to determine the best cutoff value (when the sum of sensitivity and specificity was the largest), and p exceeded the cutoff value was considered as positive. Based on the expression levels of Biomarkers in the training set, the constructed diagnostic model was used for ROC analysis, and the AUC value obtained represented the classification effect of the logistic regression model of the candidate Biomarkers on the samples of the test set, and the AUC value of the test set was the same.
