# Supplementary figures and images for: LC-MS/MS analysis reveals plasma protein signatures associated with lymph node metastasis in colorectal cancer
Source: Front Immunol. 2024 Oct 23;15:1465374. doi: 10.3389/fimmu.2024.1465374 (PMC11538601; doi:10.3389/fimmu.2024.1465374)

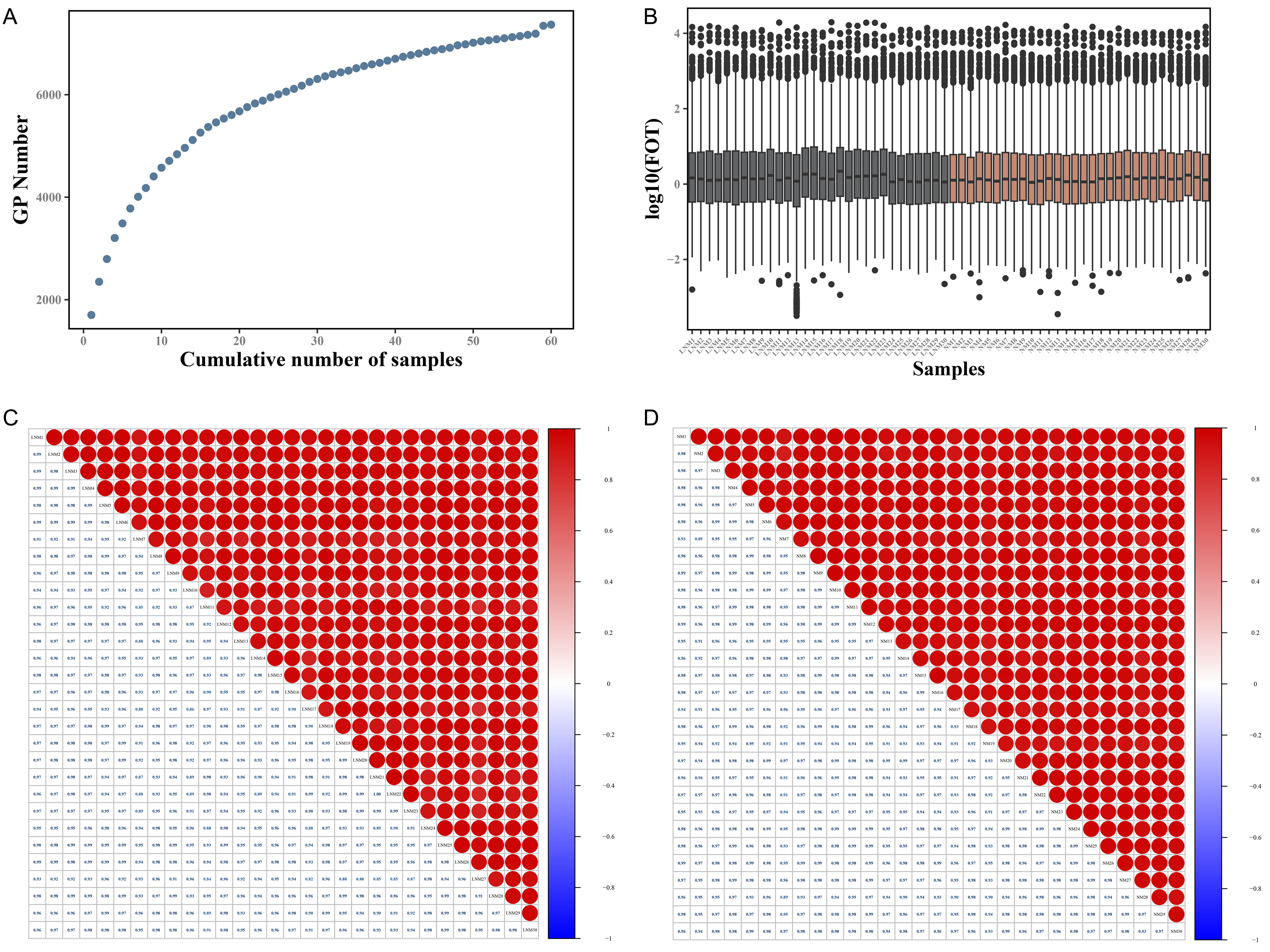

Supplement: Supplementary Figure 1 — Protein abundance and correlation analysis among samples. (A) Cumulative protein identification counts across all samples. (B) Protein Quantitative Abundance Density Distribution Chart. (C) Correlation analysis among the LNM group samples. (D) Correlation analysis among the NM group samples. [file Image1.tif]

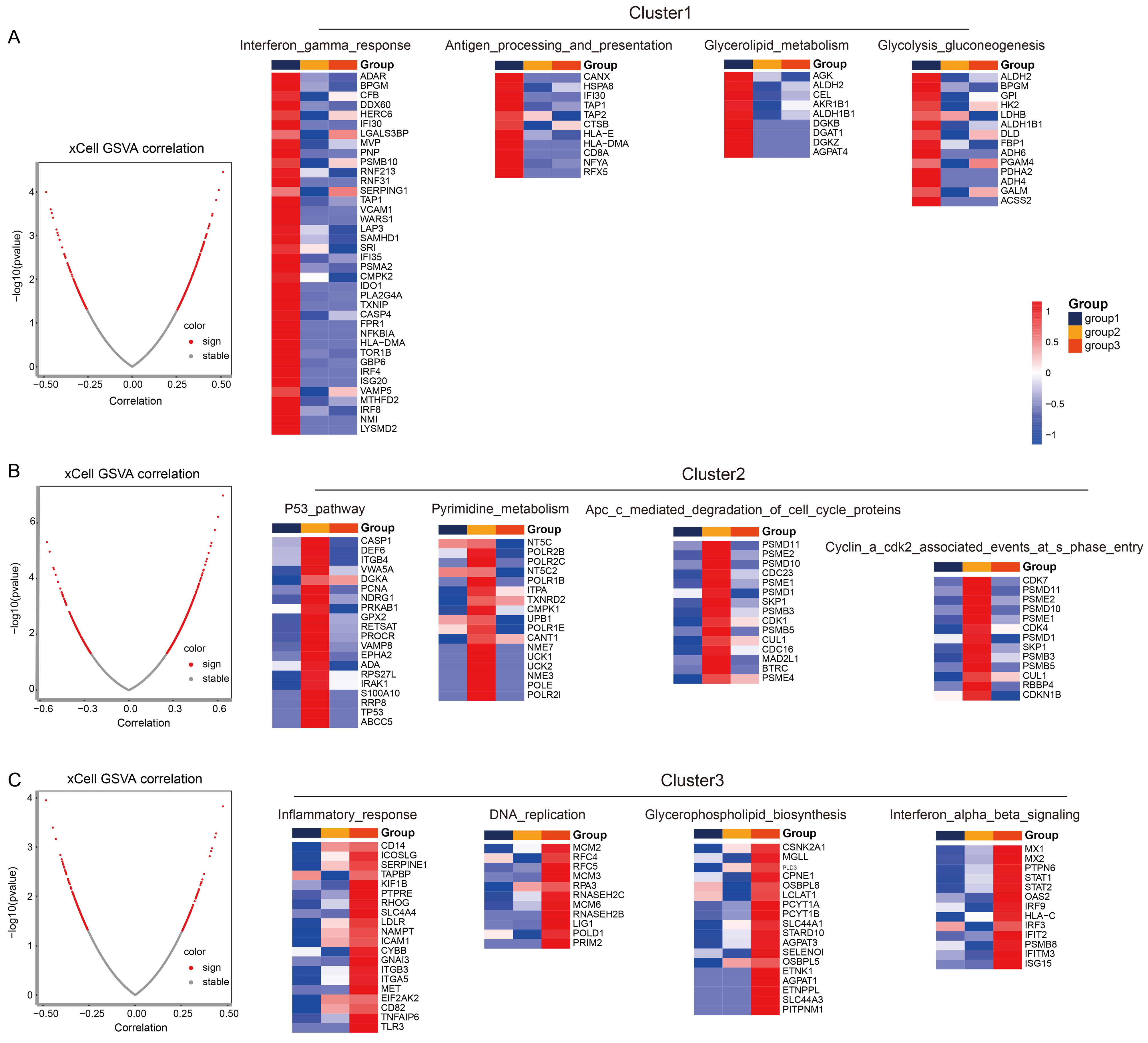

Supplement: Supplementary Figure 2 — Pathways significantly associated with immune subtyping characteristics. (A–C) Scatter plots depict the pathways exhibiting significant correlations with immune cells in immunophenotyping (A cluster 1, B cluster 2, and C cluster 3) (left). The heatmap revealed significant positive correlations between immunophenotyping and the expression of specific molecules within the pathways (right). [file Image2.tif]

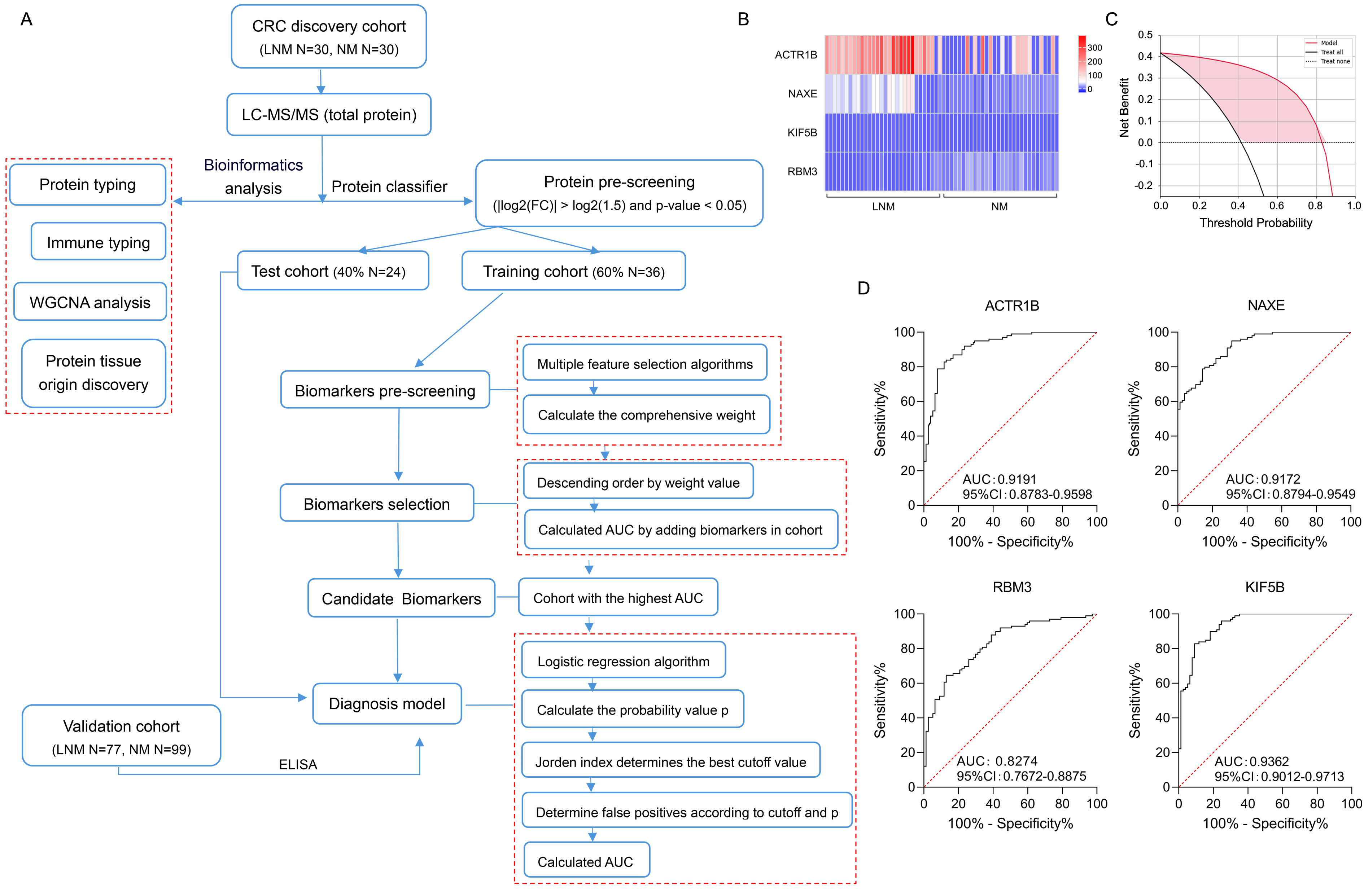

Supplement: Supplementary Figure 3 — (A) Flow chart of the study. (B) The mass spectrometry results for these four proteins. (C) DCA for assessing the clinical utility of our established protein classifier. (D) The ROC curve analysis for the four proteins detected using ELISA. [file Image3.tif]
